# The Effect of Urbanization on Population Health: Evidence From China

**DOI:** 10.3389/fpubh.2021.706982

**Published:** 2021-06-16

**Authors:** Tuan-Biao Jiang, Zi-Wei Deng, Yu-Peng Zhi, Hao Cheng, Qing Gao

**Affiliations:** ^1^School of Economics and Management, Guangxi Normal University, Guilin, China; ^2^School of Economics and Management, Guangxi Normal University, Guilin, China; ^3^Business School, Guangxi University, Nanning, China; ^4^College of Economics and Management, Nanning Normal University, Nanning, China; ^5^Graduate School, Nanning Normal University, Nanning, China

**Keywords:** economic development, urbanization, population health, panel unit root, panel threshold regression model

## Abstract

This paper explores the relationship between urbanization rate and death incidence by applying panel threshold regression model to the inland provinces of China. The empirical results highlight that there is a nonlinear single threshold effect between urbanization and population health indicators. In China's inland provinces, the negative impact of urbanization on death rate is reduced when per capita GDP exceeds the threshold, that is, the positive impact of urbanization on population health is significantly weakened. Similarly, this result can also be applied to the north provinces, while there is a no threshold effect in south. These asymmetric effects are strongly related to geographical location, historical background, economic development conditions, and health policies. Therefore, in the urbanization process, while promoting the steady development of population urbanization, the government should also increase health investment to improve the system and mechanism, formulate policies to raise health awareness, protect residents' health and reduce the waste of health resources.

## Introduction

The main purpose of this paper is to explore whether China's population health (described using death rate, DR) responds to urbanization (described as the proportion of the urban population to the total population, URB), and how it affects population health during the rapid development of urbanization. As stated in Agenda 21 for Sustainable Development of the United Nations, healthy development cannot occur without a healthy population. In the past 10 years, China's urbanization has developed rapidly, increasing from 32.93% in 2007 to 60.6% in 2019, and it is expected to reach 65.5% in 2025. It is conservatively estimated that more than 80 million new rural migrants will be added to urban areas, far exceeding that of other countries. With the continuous improvement of the urbanization rate of China's permanent resident population, the positive correlation between urbanization and population health becomes more intuitive in the development process. China has undergone profound changes in its health care system to adapt to the enormous demographic, political, economic, and sociocultural changes resulting from the rural population transfer. Thus, these changes may affect the health of the population. Investment in the health care sector has triggered a transformation in health care, which is reflected in the decline in death rate and the increase in government spending as a share of GDP over the past 13 years. There are two ways to explain why increased levels of urbanization may lead to lower death rate (or better health). First, urbanization can provide more health information and related knowledge education by improving medical services (1–3). Second, urbanization will achieve economic development by expanding infrastructure construction, creating more job opportunities through industrialization and improving the health of the population (4, 5), which is also reflected in the change in per capita disposable income. This study can benefit decision-makers by focusing on the impact of rapid urbanization on population health. In the urbanization process, the government should formulate policies to enhance health awareness among residents, increase health investment, improve the system and mechanism of healthy development, and ensure the health of residents while promoting the stable development of population urbanization.

For developing countries, the poor are at greater risk and have much worse health than the rich. As urbanization increases, increasingly more people choose to live in cities; and the living, working, eating and other habits of these people and the surrounding environment have undergone significant changes (6). Compared with rural areas, cities can provide more ecological and cultural system services, such as more parks, gardens, and playgrounds; and can also relieve life pressure through sports venues and facilities to improve health conditions (7). From the perspective of quality of life, there are fewer medical resources in the rural areas of most countries than in cities, both in terms of the number of hospitals and the number of doctors (8); and the time-spent to obtain medical services in rural areas is also higher than that in cities (9). In addition, the participation rate of medical insurance is positively correlated with the urbanization level (10). Urbanization improves the quality of life, but at the same time it requires the government to provide sufficient supply of infrastructure and medical facilities, which expands the demand for the material supply of public administration departments. Improving immediate living conditions in cities where people live, offers the best hope for reducing morbidity, death rate and health disparities and improving the quality of life and well-being (11).

While the positive correlation between urbanization and population health is intuitively evident in the process of the continuous migration of the rural population to urban areas, the effect of increasing the urbanization level on population health remains ambiguous and controversial for China. Will continued (or even excessive) migration improve the health of city dwellers? Is there a threshold effect between the two in the sample data of China? Is there a threshold effect in the sample data of southern and northern China? Therefore, this paper makes the following contributions to the above problems. First, the empirical results found a threshold effect in China's urbanization and population health issues. This threshold effect means that per capita GDP will influence the promotion effect of urbanization on population health. Second, we divided China's inland provinces into southern and northern provinces according to their geographical locations. By comparing the same relationship between the two samples, we find that there is a threshold effect of urbanization on population health in northern China, while this effect is not significant in southern China. This difference means that the urbanization in southern China is closely related to the population health situation, the government plays a significant role in the health care service system, and the medical and health situation can better meet the needs of a larger population. Moreover, although the existing research shows the regional differences in the development of Chinese urbanization and expands the trends, it mainly does so from the perspective of geographical characteristics. This study divides the urbanization and population health problems of inland provinces into two regions, and discusses their correlation and performance at different latitudes.

The reminders of the paper are structured as follows. Section Literature Review reviews the existing literature, and section The Health Utility Model With Urbanization presents the health model. Section Methodology describes the econometric approach employed in this paper. Section Data is the data description. Section Empirical Results shows the findings of the study. Section Conclusions offers concluding remarks.

## Literature Review

Urbanization and urban expansion result in urban environmental changes, and resident lifestyle changes, which can independently and synergistically lead to human health problems (12). The effects of urbanization on population health can hardly be attributed to any cause. The urban environment is an important determinant of health (13). Urbanization is a process of urban expansion caused by industrialization and economic development (14), and promotes economic growth by expanding demand (15). Industrialization intensifies the contradiction between environmental protection and economic growth and causes health problems, which reduce the harm of industrial waste gas emission to population health (16). Compared with rural areas, the positive effect of urbanization on health is reflected in the fact that cities have better employment opportunities and incomes, better educational and medical resources, and closer social ties (17, 18). Therefore, urbanization can be regarded as one of the most pervasive social and economic forces affecting economic development, population health, and ecological health (19).

Chen et al. (20) used a multilevel logistic model to study the impact of urbanization and economic development on individual health in China, and the results show that economic development has an impact on urbanization. The relationship between individual health and urbanization of different income groups presents an inverted U-shape. The effect of urbanization on population health in the eastern and central regions is significantly greater than in the western region, where urbanization increases life expectancy and improves health level of residents mainly by improving residents' health awareness, increasing medical resources, and improving the medical insurance system and public health infrastructure investment. Ademe et al. (21) found that although there may be a series of problems in the urbanization process, efficient national governance can reduce the health problems faced by the social population. Liu and Sun (22) used a logistic model to analyse the impact of the urbanization level on various diseases, and found that areas with high urbanization levels may possess better health and a better health status of the population. Liu et al. (2) found that the ratio of infectious disease-related death rate and noninfectious disease-related death rate in the process of urbanization in China continued to decline over time. Brueckner (23) stated that except for Sub Saharan Africa, the correlation between adult death rate and urbanization in other parts of the world is significantly negative; that is, the higher the level of urbanization is, the lower the death rate.

Urbanization in developing countries is usually accompanied by population migration to cities (24). Guest (4) finds that rural-urban migration leads to inequality in economic opportunities, with greater economic progress in urban areas by researching Thailand. At the same time, however, cities need to strengthen health services for young people, especially women. Sonoda (25) found that urbanization will affects women's physical health. Colvin (26) also showed that growing urbanization affects women and men in fundamentally different ways. Women and men play different roles, frequent different public areas, and face different health risks. When studying population changes, Hong et al. (27) also found that compared with urban residents, the infant survival rate, physical condition, and health-seeking behaviors of Chinese immigrants were lower.

However, the idea that urbanization is beneficial to population health is not always confirmed. Patel and Burke (28) pointed to the chaos in the urban landscape caused by the constant movement of rural residents to cities. Although many people believe that urbanization will improve the quality of life, in fact, urbanization is harmful to the health of some vulnerable groups, which also manifests in the increased incidence of endemic diseases and the possibility of epidemics expanding. As the urban population continues to grow in a short period of time, the growth rate of medical resources often falls far behind, which will produce a “crowding effect” on urban residents and have a negative impact on health (29). Srole (30) rejected the view that urban life increases the incidence of mental disorders, but urbanization may indeed result in psychological disasters for white and black children growing-up (31–33), which is evident in the slums. Eckert et al. (34) also believe that although urbanization reduces the risk of malnutrition in children, it will increase the risk of being overweight, and risk factors such as chronic diseases are more common in urban areas. Poel et al. (35) used data from the China Health and Nutrition Survey to construct urbanization indicators for research and found that urbanization increased the probability of reporting poor health. The higher the degree of urbanization is, the greater the impact will be. This finding is because the population undergoing urbanization tends to consume more cigarettes and consume more fat. Miao and Wu (36) found that the improvement of the urbanization level can lead to the reduction of high-fat diets and physical activities, reducing the health benefits brought by high income. Air and water pollution caused by industrial development will also affect population health (37, 38).

In summary, studies conducted in different countries and over different periods have yielded mixed results. In addition, it is unclear whether there is a threshold effect between urbanization and population health. Is the impact on health still positive in the urbanization process? This paper will conduct an in-depth study of the above issues.

## The Health Utility Model with Urbanization

We refer to Becker and Grossman's health demand model (39) to describe the impact of urbanization on population health. The maximum of the utility function is set to *U*(*H, Z*), where *H* is population health (described by the death rate), and *Z* is consumer production that enables utility to be obtained. *H* depends on medical care (M), nonwork time (N), and baseline status (B), with HM, HN, and HB > 0 (the subscript is the partial derivative). Theoretically speaking, health affects itself mainly in two ways. The first is that health brings happiness to consumers and then makes the utility. Second, the healthy capital stock reduces the time cost for consumers to fall ill, thus increasing their own labor income.

The lifelong health utility function of consumer can be expressed as follows:

(1) $$\begin{array}{l} U=U\left(\emptyset_{0}H_{0},\cdots \;,\emptyset_{t}H_{t},Z_{0,}\cdots \;,Z_{t}\right) \end{array}$$

Where, health (*H*) is regarded as durable goods,

*H*_0_ is the health capital stock of a consumer at birth, *H*_*t*_ is the health capital stock during period *t*, ϕ represents the health days per unit of health capital stock, and (*Z*) donates that the other goods that consumers can buy are disposable consumables. The equation of health capital in the life cycle of consumers can be described as follows:

(2) $$\begin{array}{l} H_{t+1}={\left(1-\delta_{t}\right)H}_{t}+I_{t} \end{array}$$

Here, *H*_*t*+1_ is the health capital stock of consumers in period *t* + 1; *H*_*t*_ is the health capital stock of consumers in period *t*; *I*_*t*_ represents the health capital investment of consumers in period *t*; and δ_*t*_ represents the health depreciation rate, which is related to exogenous variables such as age, living environment and income level.

The development of urbanization provides a higher level of medical services and improves the utility of health investment. However, in urbanization development process, environmental pollution, living habits, work pressure, and other external factors may weaken population health level, thus improving the depreciation rate of consumers' health capital. Therefore, it is reasonable to introduce urbanization into the healthy depreciation rate equation.

The healthy depreciation rate equation takes the form of Cropper:

(3) $$\begin{array}{l} \delta_{t}=\delta_{0}P_{t}^{\alpha }L_{t}^{\beta } \end{array}$$

Here, δ_0_ is the initial health depreciation rate, *t* is the age, *P*_*t*_ is the level of urbanization, and *L*_*t*_ is external factors such as the lifestyle of consumers.

Health investment is an important factor affecting consumers' health capital stock. The health investment function is set as the Cochrane Glass form (C-D) with a constant size, and the expression is as follows:

(4) $$\begin{array}{l} I_{t}=P_{t}^{\alpha }M_{t}^{\gamma }G_{t}^{\omega }E_{t}^{\varphi } \end{array}$$

where, *P*_*t*_ is the level of urbanization, which has a significant impact on the efficiency of consumer health investment, *M*_*t*_ is the health goods that consumers can buy, *G*_*t*_ is the basic medical services provided by the government, and *T*_*t*_ is the time consumers need to produce health. *E*_*t*_ is the other influencing factors such as consumer income.

After substitution into the equation, we obtain:

(5) $$\begin{array}{l} H_{t+1}={\left(1-\delta_{0}P_{t}^{\alpha }L_{t}^{\beta }\;\right)H}_{t}+P_{t}^{\alpha }M_{t}^{\gamma }G_{t}^{\omega }E_{t}^{\varphi } \end{array}$$

Then, we calculate the equation and obtain the partial derivative:

(6) $$\begin{array}{l} \frac{{\partial H}_{t+1}}{\partial P_{t}}=\alpha P_{t}^{\alpha -1}\left(M_{t}^{\gamma }G_{t}^{\omega }E_{t}^{\varphi }-{\delta_{0}L}_{t}^{\beta }H_{t}\right) \end{array}$$

Generally, the level of government health expenditure (*G*_*t*_) and the external factors of health depreciation(*L*_*t*_) will not change much in the short term. In conclusion, the influencing factors of the urbanization rate on the consumer health capital stock are determined by the medical expenditure level (*M*_*t*_), consumer income *E*_*t*_, government health expenditure level (*G*_*t*_), etc.

## Methodology

### Test of Gibrat's Law

This paper constructs a basic model to test whether urbanization is in line with Gibrat's law (40), and explores the factors that affect health. Referring to Yu et al. (41), Gibrat's law can be expressed as follow:

(7) $$\begin{array}{l} \frac{S_{i,t}-S_{i,t-1}}{S_{i,t-1}}=\varepsilon_{i,t} \end{array}$$

Here, *S*_*t*_ represents the urbanization situation during period *t*. ε_*i, t*_ is a normally distributed variable that follows *N*(μ, σ^2^). According to Gibrat's law, the change in the explanatory variables is independent of the independent variables. This finding shows that the healthy logarithm will follow a normal distribution, which means that in each period, the changes in growth rate should be the same magnitude for all countries in an economic organization. However, this is unrelated to initial economic development. The verification formula of Gibrat's law is defined as follows:

(8) $$\begin{array}{l} \Delta \log\left(S_{i,t}\right)=a_{i}+\beta_{i}\log\left(S_{i,t-1}\right)+\varepsilon_{i},t \end{array}$$

In this paper, the null hypothesis is β_*i*_ ≤ 0. The LLC panel unit root test and ADF-Fisher test are used to calculate β_*i*_.

### Panel Threshold Regression Model (PTRM)

Referring to the panel single threshold regression model of Hansen (42), we define *z*_*it*_ as follows: {*z*_*it*_, *URB*_*it*_, *x*_*it*_ : 1 ≤ *i* ≤ *n*, 1 ≤ *t* ≤ *T*}

Then, the following single threshold model is established:

(9) $$\begin{array}{l} z_{it}=\{\begin{array}{c} \lambda_{it}+\alpha_{1}URB_{it}+\beta_{1}^{\prime }x_{it}+\varepsilon_{it},\;if\;LPGDP_{it}\leq \gamma \;\\ \lambda_{it}+\alpha_{2}URB_{it}+\beta_{2}^{\prime }x_{it}+\varepsilon_{it},\;if\;LPGDP_{it}>\gamma \end{array} \end{array}$$

Here, *URB*_*it*_ is urbanization as the threshold variable; *z*_*it*_ is the death rate; *LGDP* is the logarithm of per capita GDP; γ is the estimated threshold value; *a*_1_ and *a*_2_ are the threshold coeffcients; *x*_*it*_ are the control variables; β_1_ and β_2_ are coeffcients of the control variables; λ_*it*_ denotes the fixed effect in different provinces under varying conditions; ε_*it*_ follows a normal distribution with a mean of 0 and a variance of σ^2^, denoted as *N*(0, σ2); and *i* and *t* represent the provinces and time, respectively.

Equation (6) can also be expressed as follows:

(10) $$\begin{array}{l} z_{it}=\lambda_{it}+\alpha_{1}URB_{it}\varphi \left(LPGDP_{it}\leq \gamma \right)+\alpha_{2}URB_{it}\varphi \\ \;\left(LPGDP_{it}>\gamma \right)+\beta^{\prime }x_{it}+\varepsilon_{it} \end{array}$$

However, there may be more turning points in applications. Hence, the multiple shapes of the threshold model can be expressed as:

(11) $$\begin{array}{l} z_{it}=\{\begin{array}{cc} \;\lambda_{it}+\alpha_{1}URB_{it}+\beta_{1}^{\prime }x_{it}+\varepsilon_{it}, & if\;LPGDP_{it}\leq \gamma_{1}\\ \lambda_{it}+\alpha_{2}URB_{it}+\beta_{2}^{\prime }x_{it}+\varepsilon_{it}, & if\;\gamma_{1}<LPGDP_{it}\leq \gamma_{2}\\ \;\lambda_{it}+\alpha_{3}URB_{it}+\beta_{3}^{\prime }x_{it}+\varepsilon_{it},\; & \;if\;LPGDP_{it}>\gamma_{2} \end{array}\; \end{array}$$

The regression model above can also transform into the following formulation:

(12) $$\begin{array}{l} z_{it}=\mu_{it}+\alpha_{1}URB_{it}\varphi \left(LPGDP_{it}\leq \gamma_{1}\right)+\alpha_{2}URB_{it}\varphi \\ \;\left(\gamma_{1}<LPGDP_{it}\leq \gamma_{2}\right)+\alpha_{3}URB_{it}\varphi \left(LGPDP_{it}>\gamma_{2}\right)\\ \;+\beta^{\prime }x_{it}+\varepsilon_{it} \end{array}$$

Here, γ_1_ and γ_2_ are threshold values (γ_1_ < γ_2_). Similarly, more threshold regression models for turning points can be derived in the same way.

## Data

This sample used the annual data of China's inland provinces from 2007 to 2019 (Tibet's statistical data are seriously missing, so they are deleted) including a total of 390 annual entries. Since the population health and urbanization data were collected from staring in 2007, the sample coverage period began in 2007. Data sources are the China Statistical Yearbook, the National Bureau of Statistics, the National Research Network Statistical Database, and the China Economic and Social Development Statistical Database. Previous studies believe that death rate (*DR*) can directly reflect the medical level and health level of residents in a country or region, which is important for social development and family planning policy. This paper uses the urban population to total population ratio (*URB*) to measure the level of urbanization, and uses logarithm of per capita GDP (*LPGDP*) as the threshold variable. Generally, a large number of rural people will flow to cities, the urbanization ratio will increase, the urban-rural integration will accelerate, the population structure will change, and the service object and content of the medical structure will gradually change. Empirical studies show that the improvement of the urbanization level has a positive impact on population health. The government's investment in health care is also an expensive expenditure that which cannot guarantee the expected return. This urges people to study the threshold effect of urbanization (*URB*).

Six control variables are introduced in this study. The first is environmental pollution (*EP*), measured by industrial sulfur dioxide emissions. In the fuel combustion process and the production process, enterprises will emit sulfur dioxide into the atmosphere, which will not only bring environmental problems such as acid rain, but also may cause allergic reaction when excessive intake into the human body harms health. Thus, the emission of sulfur dioxide will affect people's health. The second is per capita disposable income (*CI*), which is the most important determinant of consumer spending and usually used to measure the change in living standards (43, 44). Generally, residents with a higher level of personal disposable income will be able to pay more for medical care when they face health problems (45). To ensure the accuracy of the statistical results, we used the logarithm of this index in the empirical process. The third is the service sector share (*SEV*), measured by the share of total with respect services to GDP. The proportion of the service industry is usually regarded as an important factor affecting the health care industry. There are many factors leading to an increase in *SEV*, such as the adjustment of the industrial division of labor and increased costs. Overall, the diversification of the service industry structure will impact population health (46, 47). To ensure the accuracy of the statistical results, we used the logarithm of this index in the empirical process. The fourth is population density (*PD*), which refers to the number of people per unit of land area and is an important indicator reflecting the population distribution. Excessive population density may lead to intensified job competition, cause excessive psychological pressure and harm physical health. Furthermore, it will also affect the air and environment, resulting in decreased cardiopulmonary functioning and population health (48). The fifth is the government spending share (*GOV*), measured by government spending as a share of GDP. In 1994, to improve people's health and accelerate economic development, China began to implement medical system reform to improve the effectiveness of government health spending on health welfare production and gain. The higher the proportion of health expenditures is, the greater the government's investment in national health, which can effectively affect the health situation of residents. The sixth is educational attainment (*EDU*), measured by the proportion of the population with a college degree or above. Yasuhiko Kubota, a visiting researcher at the University of Minnesota in the United States, found that education is more likely to affect health than income through a follow-up investigation for more than 20 years; and the higher one's education is, the less likely they are to suffer from cardiovascular and cerebrovascular diseases (49). This shows that the level of education can affect the health of the population.

Table 1 divides the statistical data into two subsamples of 30 inland provinces, 15 northern provinces and 15 southern provinces, based on the geographical dividing line Qinling and Huaihe River in China. As Table 1 shows, the average death rate in China is higher at 6.034%, and that in southern provinces (6.195%) is higher than that in the northern provinces (5.873%). This may be related to the differences in living environments and habits. The average urbanization rate of southern provinces (54.667%) is slightly lower than the average urbanization rate of northern provinces (55.806%), both of which are not much different from the overall urbanization rate. This may be due to the differences in the geographical location and climate characteristics of the south. As long as the northern regions focus on industrial development, the southern provinces will enjoy better agricultural development, which is reflected in a higher proportion of rural population, that is, urbanization is relatively low. Since the development of industry is the main economic development mode in the north, the sulfur dioxide emissions of the northern provinces (52.941) are much higher than those of the south provinces (44.831). In addition, the southern provinces far outperformed their northern counterparts in terms of per capita disposable income, population density and government spending as a share of GDP. Jarque-Bena test results show that all data sequences obey a normal distribution.

**Table 1:** Descriptive statistics of the variables.

		**Obs**	**Mean**	**Std**.**Dev**.****	**Minimum**	**Maximum**	**Skewness**	**Kurtosis**
Inside provinces	DR	390	6.034	0.752	4.210	7.570	0.001	0.080
	URB	390	55.236	13.293	28.240	89.600	0.000	0.072
	EP	390	48.886	37.818	0.032	162.865	0.000	0.329
	CI	390	41.925	7.040	24.898	68.256	0.045	0.491
	SEV	390	−0.839	0.201	−1.251	−0.180	0.000	0.009
	PD	390	458.746	677.876	7.637	3853.968	0.000	0.000
	GOV	390	5.360	0.879	2.435	7.365	0.000	0.701
	EDU	390	0.120	0.073	0.012	0.505	0.000	0.000
	LPGDP	390	10.592	0.564	8.841	12.009	0.254	0.483
North provinces	DR	195	5.873	0.741	4.260	7.500	0.063	0.047
	URB	195	55.806	13.161	32.250	86.600	0.000	0.265
	EP	195	52.941	44.275	0.032	162.865	0.000	0.000
	CI	195	39.404	7.221	24.898	67.031	0.000	0.028
	SEV	195	−0.852	0.229	−1.251	−0.180	0.000	0.050
	PD	195	346.220	397.132	7.637	1382.301	0.000	0.038
	GOV	195	5.173	0.860	2.435	6.894	0.011	0.739
	EDU	195	0.136	0.084	0.038	0.505	0.000	0.000
	LPGDP	195	10.617	0.516	9.244	12.009	0.850	0.960
South provinces	DR	195	6.195	0.729	4.210	7.570	0.001	0.927
	URB	195	54.667	13.434	28.240	89.600	0.000	0.100
	EP	195	44.831	29.569	0.652	117.600	0.063	0.001
	CI	195	44.446	5.870	32.687	68.256	0.460	0.202
	SEV	195	−0.825	0.169	−1.246	−0.318	0.015	0.412
	PD	195	571.273	859.204	117.767	3853.968	0.000	0.000
	GOV	195	5.547	0.859	2.523	7.365	0.000	0.346
	EDU	195	0.104	0.056	0.012	0.340	0.000	0.000
	LPGDP	195	10.568	0.609	8.841	11.966	0.222	0.235

## Empirical Results

We perform a panel unit root test to investigate whether the population health data of inland provinces in China are consistent with Gibra's law. Due to the poor performance of the IPS test in the samples in this paper, the Levin, Lin and Chu (LLC) test and Fisher-ADF test are considered to be effective methods for testing the existence of unit roots according to the research results of Shiller and Perron (50, 51). As Table 2 shows, there is no unit root of the death rate, which does not comply with Gibrat's law. Furthermore, to avoid the existence of a false regression, the stability of all variables should be maintained before the use of the PTRM. The panel unit root tests of the LLC and Fisher-ADF show that the variables are all significant at least at the 10% level. Therefore, we proceed to analyse the PTRM.

**Table 2:** Panel unit root tests.

	**Variables**	**Panel augmented dickey-fuller test**			
		**Levin-Lin-Chu**		**Fisher-ADF**	
		***t-*statistic**	***p*-value**	**Statistic**	***p-*value**
Inside provinces	*DR*	−9.627	0.000	157.144	0.000
	*URB*	−5.716	0.000	153.356	0.000
	*EP*	−4.792	0.000	85.590	0.017
	*CI*	−22.422	0.000	77.036	0.068
	*SEV*	−4.920	0.000	157.930	0.000
	*PD*	−4.586	0.000	375.614	0.000
	*GOV*	−8.804	0.000	187.502	0.000
	*EDU*	−8.953	0.000	160.128	0.000
	*LPGDP*	−3.001	0.001	86.875	0.013
North provinces	*DR*	−5.681	0.000	67.295	0.000
	*URB*	−3.283	0.001	64.222	0.000
	*EP*	−2.808	0.003	47.152	0.024
	*CI*	−11.105	0.000	48.704	0.017
	*SEV*	−2.852	0.002	83.672	0.000
	*PD*	−15.691	0.000	128.126	0.000
	*GOV*	−8.867	0.000	99.592	0.000
	*EDU*	−6.684	0.000	66.387	0.000
	*LPGDP*	−2.138	0.016	45.199	0.037
South provinces	*DR*	−8.080	0.000	110.540	0.000
	*URB*	−5.236	0.000	74.115	0.000
	*EP*	−4.350	0.000	47.936	0.020
	*CI*	−6.123	0.000	71.047	0.000
	*SEV*	−2.923	0.002	69.633	0.000
	*PD*	−5.535	0.000	183.720	0.000
	*GOV*	−4.516	0.000	67.454	0.000
	*EDU*	−5.783	0.000	79.093	0.000
	*LPGDP*	−4.358	0.000	70.607	0.000

The results in Table 3 present an optimal level of urbanization for the full sample of 30 inland provinces and for the southern and northern provinces in China. Overall, the single threshold for logarithm of per capita GDP is 10.237, which is significant at the 5% significance level; and the *F-statistic* is 26.740. Table 4 shows that there is a significant negative correlation between *URB* and *DR* when China's logarithm of per capita GDP is lower than 10.237, with an estimated coefficient of −0.036. The negative relationship between urbanization and death rate becomes weaker when the logarithm of per capita GDP is greater than a certain limit threshold. This indicates that there is a single threshold effect, which makes the model show an asymmetric non-linear relationship with the inflection point.

**Table 3:** Test for threshold between Urbanization and Population health.

	**Single threshold effect test**			**Double threshold effect test**		
	**Threshold value**	**F-statistics**	***p*-value**	**Threshold value**	**F-statistics**	***p*-value**
**ALL**						
Inside provinces	10.237^**^	26.740	0.050	10.841	11.200	0.364
North provinces	10.823^*^	21.280	0.054	11.412	14.590	0.130
South provinces	10.237	14.260	0.256	10.881	10.720	0.248

***, **, and * respectively indicates significance at the 1, 5, and 10% level.

**Table 4:** Estimated coefficients of Urbanization and Population health.

**Regions**	**Coefficient**	**Estimated value**	***p*-value**	**Std.Dev.**
Inside provinces	${\hat{\alpha }}_{1}$	−0.036^***^	0.001	0.011
	${\hat{\alpha }}_{2}$	−0.031^***^	0.004	0.011
North provinces	${\hat{\alpha }}_{1}$	−0.083^***^	0.000	0.019
	${\hat{\alpha }}_{2}$	−0.076^***^	0.000	0.019
South provinces	${\hat{\alpha }}_{1}$	−0.002	0.876	0.011
	${\hat{\alpha }}_{2}$	0.002	0.863	0.011

***, **, and * respectively indicates significance at the 1, 5, and 10% level.

For northern provinces, the single threshold for the logarithm of per capita GDP is 10.823, which is significant at the 10% significance level and the *F-*statistic is 21.280. Additionally, Table 4 shows that there is a significant negative correlation between *URB* and *DR* when the logarithm of per capita GDP of northern provinces is below the threshold value of 10.823 with an estimated coefficient of −0.083. The coefficient of DR changes to −0.076 when the logarithm of per capita gdp exceeds 10.823. This means that the increase in the urbanization level can effectively reduce the death rate of northern provinces and has a positive effect on population health. The results indicate that there is still a single threshold effect for the northern provinces.

In contrast, in southern provinces, the single threshold for logarithm of per capita GDP is 10.237 with an *F*-statistics of 14.260, but the results did not pass the significance test. As Table 4 shows, when the logarithm of per capita GDP of southern provinces is 10.237, there is no significant correlation between *URB* and *DR*. The results show that there is no single threshold effect in this model for the southern provinces, which has not been found in previous studies. This might relate to different geographical distribution and cultural traditions. Regarding population and economic growth, the urbanization process of northern provinces in China is relatively slow, and the economic development level in the north is lower than that of the south. Health problems caused by population migration can be solved by government health investment spending due to the better economic conditions and the popularization of relevant health knowledge in southern China. In other words, the growth rate of the population health level in southern China can keep up with the pace of urbanization development, which is embodied in the fact that there is no single threshold effect between *DR* and *URB*.

**Figure 1:**
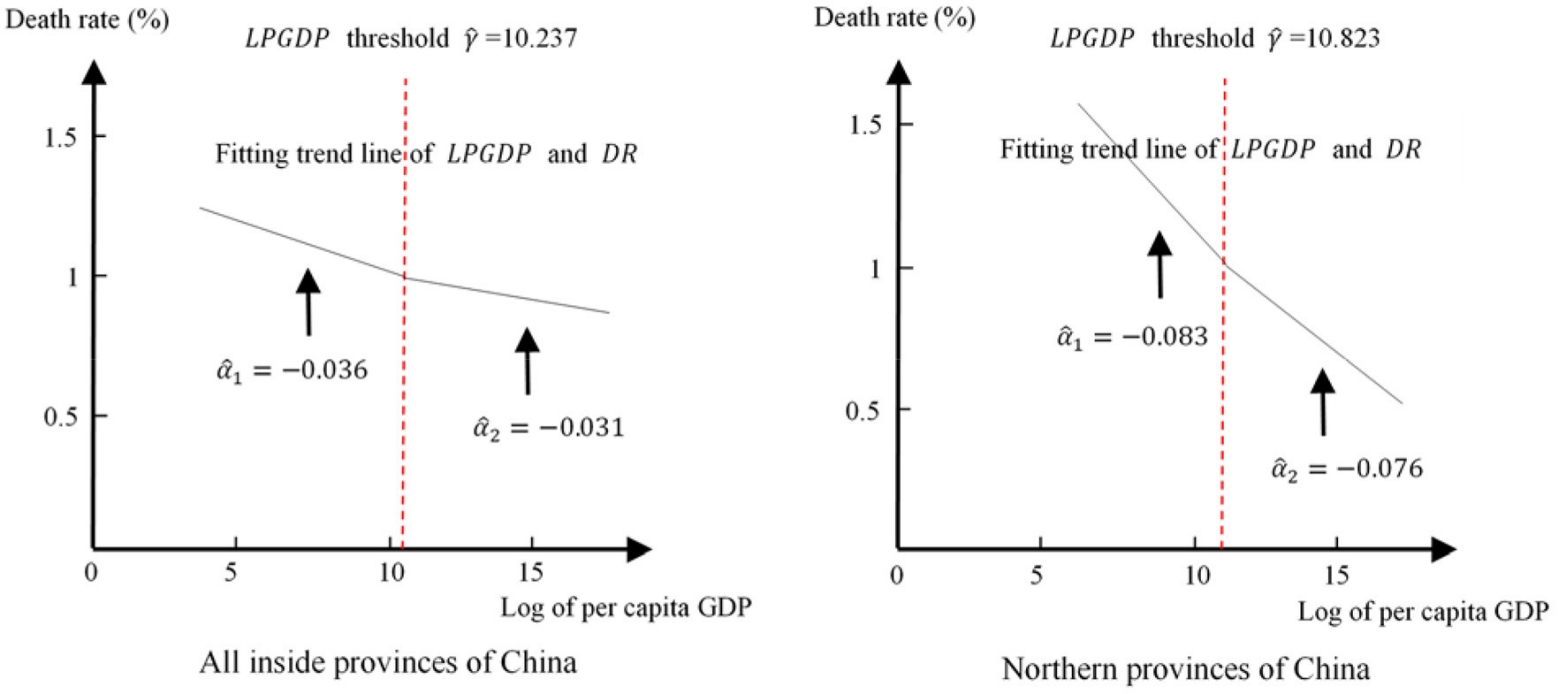
Simulation of the relationship between urbanization and population health.

Whether in China or in the northern provinces, urbanization has played a role in promoting population health, but the relationship between urbanization and population health will not be the same in a specific time. In this paper, the explained variable *DR* has an inverse relationship with population health, that is, the lower the death rate, the higher the health of the population, and vice versa. Figure 1 shows that for China, when the logarithm of per capita GDP is lower than 10.237, the coefficient of *DR* is −0.036, and the coefficient of *DR* changes to −0.031 when the logarithm of per capita GDP crosses the threshold. Similarly, in northern provinces, when the logarithm of per capita GDP is lower than 10.823, the coefficient is −0.083, and it changes to −0.076 when the logarithm of per capita GDP crosses the threshold. This indicates that in China and the northern provinces, the impact of urbanization on population health has a significant single threshold effect based on per capita GDP, which means that when per capita GDP exceeds a certain threshold, the role of urbanization in promoting population health is weakened.

For the thresholds of China and northern provinces, we can further confirm the impact of each control variable on population health in Table 5. As Table 5 shows, in the full sample and for northern provinces, environmental pollution *EP*, government spending share *GOV*, and population density *PD* can significantly affect death rate *DR* at a significant level of at least 10%, while other control variables are not always significant. According to the coefficients in Table 5, *EP* is negatively correlated with *DR* in China and north region, but it is not significant in south. The possible reason is that industry is one of the important sources of environmental pollution. It is labor-intensive and has a population gathering effect, which makes environmental pollution and death rate negatively correlated. Since the heavy industry is concentrated in northern China, the southern manufacturing and service industries have developed well, and the degree of environmental pollution is relatively low. In addition, *GOV* and *PD* have positive and negative effects on DR, respectively. Furthermore, the coefficient of per capita disposable income *CI* in the northern region can also pass the test at the 5% significance level. Increasing per capita disposable income will promote consumption, which may lead to some diseases such as obesity, and will cause higher death rate in turn.

**Table 5:** Estimated coefficients of the control variables.

**Regions**	**Variable**	**Coefficient**	**Estimated value**	***p*-value**	**Std.Dev.**
Inside provinces	EP	${\hat{\beta }}_{1}$	−0.002^*^	0.096	0.001
	CI	${\hat{\beta }}_{2}$	0.007	0.130	0.005
	GOV	${\hat{\beta }}_{3}$	0.204^**^	0.010	0.079
	SEV	${\hat{\beta }}_{4}$	0.349	0.196	0.269
	PD	${\hat{\beta }}_{5}$	−0.003^***^	0.000	0.000
	EDU	${\hat{\beta }}_{6}$	1.630^*^	0.079	0.926
North provinces	EP	${\hat{\beta }}_{1}$	−0.003^**^	0.039	0.002
	CI	${\hat{\beta }}_{2}$	0.018^**^	0.013	0.007
	GOV	${\hat{\beta }}_{3}$	0.426^***^	0.003	0.140
	SEV	${\hat{\beta }}_{4}$	0.202	0.651	0.445
	PD	${\hat{\beta }}_{5}$	−0.002^***^	0.001	0.001
	EDU	${\hat{\beta }}_{6}$	1.057	0.463	1.437
South provinces	EP	${\hat{\beta }}_{1}$	−0.000	0.853	0.001
	CI	${\hat{\beta }}_{2}$	−0.016^**^	0.035	0.007
	GOV	${\hat{\beta }}_{3}$	0.014	0.866	0.083
	SEV	${\hat{\beta }}_{4}$	0.610^**^	0.033	0.284
	PD	${\hat{\beta }}_{5}$	−0.002^***^	0.000	0.000
	EDU	${\hat{\beta }}_{6}$	0.008	0.994	1.158

***, **, and * respectively indicates significance at the 1, 5, and 10% level.

Considering the irreplaceability of *DR* and *URBAN*, to obtain a more reliable conclusion, following the same method in the references, we select three new control variables to conduct a robustness test. The first new control variable is *GDP* (logarithm, *LGDP*). Studies have shown that human health is related to per capita disposable income (*CI*) and has a similar cointegration relationship with GDP, often as a direct economic indicator affecting population health (52). The second new control variable is the consumer price index (CPI), which reflects the changes in the price level of consumer goods and services usually purchased by families, and can measure the changes in the overall economic level of society (53). The third control variable is financial development level (*FINA*), which is measured by the percentage of financial institutions' deposits and loans to GDP at the end of the year, and can affect population health by affecting economic development.

Table 6 shows that after adding the new control variables *LGDP*, consumer price index *CPI*, and financial development level (*FINA*), the single threshold effect still exists in the entire sample of inland provinces and northern provinces of China, and the threshold value does not change significantly. However, there is no threshold effect in southern provinces. Although the threshold value did not change significantly, the *p*-values of the entire sample decreased from 0.050 to 0.042, and increased from 0.054 to 0.064 in northern provinces. This indicates that in China, the threshold effect of urbanization on death rate becomes more significant after the addition of the control variables *LGDP*, *CPI*, and *FINA*, that is, before the logarithm of per capita GDP reaches the certain threshold, there is a significant negative correlation between URB and DR, and urbanization can effectively improve the health of the population. When the logarithm of per capita GDP exceeds 10.237, the health-promoting effect of urbanization will become significantly weaker. This is consistent with the results in Tables 3, 4, so the conclusion of this paper is valid. In Table 7, the regression coefficients of the control variables shows that environmental pollution (*EP*), population density (*PD*), and *GDP* have significant impacts on the death rate (*DR*), while the other control variables (especially education *EDU*) are not always significant.

**Table 6:** Robustness check.

	**Single threshold effect test**			**Double threshold effect test**		
	**Threshold value**	**F-statistics**	***p*-value**	**Threshold value**	**F-statistics**	***p*-value**
**ALL**						
Inside province	10.237^**^	26.080	0.042	10.835	5.700	0.774
North provinces	10.823^*^	21.610	0.064	11.379	11.370	0.198
South provinces	10.237	15.970	0.182	10.836	7.120	0.560

***, **, and * respectively indicates significance at the 1, 5, and 10% level.

**Table 7:** Estimated coefficients of the control variables.

**Region**	**Variable**	**Coefficient**	**Estimated value**	***p*-value**	**Std.Dev.**
Inside provinces	EP	${\hat{\beta }}_{1}$	−0.003^***^	0.008	0.001
	CI	${\hat{\beta }}_{2}$	0.010^**^	0.128	0.006
	GOV	${\hat{\beta }}_{3}$	−0.152	0.287	0.143
	SEV	${\hat{\beta }}_{4}$	0.818^***^	0.005	0.287
	PD	${\hat{\beta }}_{5}$	−0.003^***^	0.000	0.000
	EDU	${\hat{\beta }}_{6}$	0.322	0.751	1.012
	CPI	${\hat{\beta }}_{7}$	−0.001	0.924	0.010
	LGDP	${\hat{\beta }}_{8}$	0.960^***^	0.001	0.284
	FINA	${\hat{\beta }}_{9}$	−0.138^*^	0.079	0.078
North provinces	EP	${\hat{\beta }}_{1}$	−0.004^**^	0.013	0.002
	CI	${\hat{\beta }}_{2}$	0.016	0.115	0.010
	GOV	${\hat{\beta }}_{3}$	0.072	0.747	0.223
	SEV	${\hat{\beta }}_{4}$	0.824^*^	0.084	0.474
	PD	${\hat{\beta }}_{5}$	−0.003^***^	0.000	0.001
	EDU	${\hat{\beta }}_{6}$	0.440	0.780	1.573
	CPI	${\hat{\beta }}_{7}$	0.019	0.281	0.018
	LGDP	${\hat{\beta }}_{8}$	0.777^*^	0.056	0.404
	FINA	${\hat{\beta }}_{9}$	−0.056	0.627	0.116
South provinces	EP	${\hat{\beta }}_{1}$	−0.001	0.438	0.011
	CI	${\hat{\beta }}_{2}$	−0.011	0.206	0.439
	GOV	${\hat{\beta }}_{3}$	−0.356^*^	0.058	0.111
	SEV	${\hat{\beta }}_{4}$	1.056^***^	0.001	0.001
	PD	${\hat{\beta }}_{5}$	−0.002^***^	0.002	0.008
	EDU	${\hat{\beta }}_{6}$	−1.717	0.166	0.186
	CPI	${\hat{\beta }}_{7}$	−0.021^*^	0.068	0.315
	LGDP	${\hat{\beta }}_{8}$	1.282^***^	0.004	0.001
	FINA	${\hat{\beta }}_{9}$	−0.304^***^	0.007	1.234

***, **, and * respectively indicates significance at the 1, 5, and 10% level.

## Conclusions

This paper performs a panel unit root test to examine whether Gibrat's law is applied to China's urbanization. Table 2 shows that the stationary hypothesis of death rate cannot be rejected, which suggests that health indicators follow a random walk and urbanization is an important variable effect on population health. Subsequently, this paper uses panel data to explore the threshold effects of urbanization on population health in China and the northern and southern provinces of China by using the PTRM. In our study, urbanization is defined as the proportion of the urban population with respect to the total population (*URB*), and we find that 10.237 and 10.823 serve as the threshold rate for logarithm of per capita GDP in China and northern provinces, respectively. When logarithm of per capita GDP falls below this level, the increase in urbanization and economic will contribute to improved population health. Once this level is exceeded, the negative relationship of urbanization on death rate is significantly weaker, that is, the health-promoting effect of urbanization is greatly reduced. However, there is no threshold effect in the South. These findings provide valuable insights for policy makers on how to equitably deliver health services to improve the health status of populations in the face of rapid urbanization. Urbanization plays a positive role in promoting the health of the population, and the government can improve the health level of residents by promoting urbanization, so that urbanization will benefit people. However, urbanization should not be seen as a solution to health problems, but should be accompanied by effective health policies to avoid wasting medical resources in the social development process.

## Data Availability Statement

The original contributions presented in the study are included in the article/supplementary material, further inquiries can be directed to the corresponding author/s.

## Author Contributions

T-BJ: conceptualization and methodology. Z-WD: software and writing-reviewing. Y-PZ: visualization and data curation. HC: writing-original draft preparation. QG: editing and investigation. All authors contributed to the article and approved the submitted version.

## Conflict of Interest

The authors declare that the research was conducted in the absence of any commercial or financial relationships that could be construed as a potential conflict of interest.

## References

[1] Duhl LJ. Health and urban development: IV. Urbanization and human needs. Am J Publ Health Nations Health. (1964) 54:721. 10.2105/AJPH.54.5.721 PMC1254855 14153272

[2] Liu M Liu X Huang Y Ma Z Bi J. Epidemic transition of environmental health risk during China's urbanization. Sci Bull. (2017) 62:92–8. 10.1016/j.scib.2016.12.004 36659489

[3] Godfrey R Julien M. Urbanisation and health. Clin Med. (2005) 5:137–41. 10.7861/clinmedicine.5-2-137 PMC4952865 15847005

[4] Guest P. Urbanization and its implications for health services. J Popul Soc Stud. (1998) 7:21–52. 12294262

[5] Yusof K Kwai-Sim L. Urbanization and its effects on health in squatter areas (with special reference to Kuala Lumpur, Malaysia). J Hum Ergol. (1990) 19:171–84. 10.11183/jhe1972.19.171 2130090

[6] Phillips D.R. Urbanization and human health. Parasitology. (1993) 106:S93–107. 10.1017/S0031182000086145 8488075

[7] Kabisch N Bosch M Lafortezza R. The health benefits of nature-based solutions to urbanization challenges for children and the elderly - a systematic review. Environ Res. (2017) 159:362–73. 10.1016/j.envres.2017.08.004 28843167

[8] Jing Z. The impact of water quality on health: evidence from the drinking water infrastructure program in rural China. J Health Econ. (2012) 31:122–34. 10.1016/j.jhealeco.2011.08.008 22019013

[9] Grumbach K Vranizan K Bindman AB. Physician supply and access to care in urban communities. Health Affairs. (1997) 16:71–86. 10.1377/hlthaff.16.1.71 9018945

[10] Liu GG Wu X Peng C Fu AZ. Urbanization and health care in rural China. Contemp Econ Pol. (2003) 21:11–24. 10.1093/cep/21.1.11

[11] Vlahov D. Urban as a determinant of health. J Urban Health. (2007) 84:16–26. 10.1007/s11524-007-9169-3 PMC1891649 17356903

[12] Li X Song J Lin T Dixon J Zhang G Ye H. Urbanization and health in China, thinking at the national, local and individual levels. Environ Health. (2016) 15:S32. 10.1186/s12940-016-0104-5 26961780 PMC4895783

[13] Ramkrishnan NR. The effects of urbanization on administrative aspects of urban health services. Indian J Publ Health. (1968) 12:85–6. 5717336

[14] Patil Rajan R. Urbanization as a determinant of health: a socioepidemiological perspective. Soc Work Publ Health. (2014) 29:335–41. 10.1080/19371918.2013.821360 24871771

[15] Liu TY Su CW Jiang XZ. Is economic growth improving urbanisation? A cross-regional study of China. Urban Stud. (2015) 52:1883–98. 10.1177/0042098014540348

[16] Dong H Xue M Xiao Y Liu Y. Do carbon emissions impact the health of residents? Considering China's industrialization and urbanization. Sci Tot Environ. (2021) 758:143688. 10.1016/j.scitotenv.2020.143688 33338785

[17] Moore M Gould P Keary BS. Global urbanization and impact on health. Int J Hyg Environ Health. (2003) 206:269–78. 10.1078/1438-4639-00223 12971682

[18] Monda KL Gordon-Larsen P Stevens J Popkin BM. China's transition: the effect of rapid urbanization on adult occupational physical activity. Soc Sci Med. (2007) 64:858–70. 10.1016/j.socscimed.2006.10.019 17125897 PMC2753984

[19] Wu JJ Oueslati W. How does urbanization affect the economy and the environment? Policy challenges and research needs. Int Rev Environ Resour Econ. (2016) 10:1–35. 10.1561/101.00000081

[20] Chen H Liu Y Li Z Xue D. Urbanization, economic development and health: evidence from China's labor-force dynamic survey. Int J Equity Health. (2017) 16:207. 10.1186/s12939-017-0705-9 29187257 PMC5707809

[21] Ademe AS Biadgilign S Yesigat H. Good governance, public health expenditures, urbanization and child undernutrition Nexus in Ethiopia: an ecological analysis. BMC Health Serv Res. (2019) 19:40. 10.1186/s12913-018-3822-2 30646917 PMC6334413

[22] Liu GF Sun MP Wang ZY Jian WY. Association analysis between urbanization and non-communicable diseases and health-related behavior. Beijing Da Xue Xue Bao Yi Xue Ban. (2016) 48:478–82. 10.3969/j.issn.1671-167X.2016.03.018 27318911

[23] Brueckner M. Adult mortality and urbanization: examination of a weak connection in sub-Saharan Africa. World Dev. (2019) 122, 184–98. 10.1016/j.worlddev.2019.05.019

[24] Hou B James N James B Marshall A. Are cities good for health? A study of the impacts of planned urbanization in China. Int J Epidemiol. (2019) 4:1083–90. 10.1093/ije/dyz031 30887030

[25] Sonoda K. Urbanization and new health indicators. J Hum Ergol. (1990) 19:233. 10.11183/jhe1972.19.233 2130095

[26] Colvin ML. Women's health and the world's cities. J Evid Inform Soc Work. (2015) 12:1–3. 10.1080/15433714.2012.759471

[27] Hong Y Li X Stanton B. Too costly to be ill: health care access and health seeking behaviors among rural-to-urban migrants in China. World Health Popul. (2006) 8:22–34. 10.12927/whp.2006.18280 18277099 PMC2249561

[28] Patel RB Burke TF. Global health: urbanization - an emerging humanitarian disaster. N Engl J Med. (2009) 361:741–3. 10.1056/NEJMp0810878 19692687

[29] Qin XZ Li LX Hsieh Chee-Ruey. Too few doctors or too low wages? Labor supply of health care professionals in China. China Econ Rev. (2013) 24:150–64. 10.1016/j.chieco.2012.12.002

[30] Srole L. Urbanization and mental health: a reformulation. Psychiatr Q. (1972) 46:449–60. 10.1007/BF01561981 4661279

[31] Ventriglio A Torales J Castaldelli-Maia JM De Berardis D Bhugra D. Urbanization and Emerging Mental Health Issues. CNS Spectr. (2020) 26:1–20. 10.1017/S1092852920001236 32248860

[32] Turan MT Besirli A. Impacts of urbanization process on mental health. Anadolu Psikiyatri Dergisi. (2008) 9:238–43. 10.5801/ncn.v14i2.585

[33] Vasoontara Y Caldwell BK Lim LY Seubsman S Sleigh AC. Lifecourse urbanization, social demography, and health outcomes among a national cohort of 71,516 adults in Thailand. Int J Popul Res. (2011) 2011:464275. 10.1155/2011/464275 22428087 PMC3303129

[34] Eckert S Kohler S. Urbanization and health in developing countries: a systematic review. World Health Popul. (2014) 15:7. 10.12927/whp.2014.23722 24702762

[35] Poel E O'Donnell O Doorslaer EV. Is there a health penalty of China's rapid urbanization? Health Econ. (2012) 21:367–85. 10.1002/hec.1717 21341344

[36] Miao J Wu X. Urbanization, socioeconomic status and health disparity in China. Health Place. (2016) 42:87–95. 10.1016/j.healthplace.2016.09.008 27750075

[37] Wang X Smith KR. Secondary benefits of greenhouse gas control: health impacts in China. Environ Sci Technol. (1999) 33:3056–61. 10.1021/es981360d

[38] Zheng SQ Kahn ME. Understanding China's urban pollution dynamics. J Econ Literat. (2013) 51:731–72. 10.1257/jel.51.3.731

[39] Grossman M. On the concept of health capital and the demand for health. J Polit Econ. (1972) 80:223–55. 10.1086/259880

[40] Gibrat R. Les Inégalites Economique. Pairs: Librairie du Recueil Sirey (1931).

[41] Yu J Chen R Fang J Guo H. Electronic information industry, clustering and growth: empirical study of the Chinese enterprises. Chinese Manage Stud. (2013) 7:172–93. 10.1108/CMS-Sep-2011-0083

[42] Hansen BE. Threshold effects in non-dynamic panels: estimation, testing, and inference. J Econometr. (1999) 93:345–68. 10.1016/S0304-4076(99)00025-1

[43] Bean LH. Relation of disposable income and the business cycle to expenditures. Rev Econ Stat. (1946) 28:199–207. 10.2307/1925416

[44] Friend I. Relationship between consumers' expenditures, savings, and disposable income. Rev Econ Stat. (1946) 28:208–15. 10.2307/1925417

[45] Ettner SL. New evidence on the relationship between income and health. J Health Econ. (1996) 15:67–85. 10.1016/0167-6296(95)00032-1 10157429

[46] Rathore K Shahid R Ali K. Factors affecting service sector's contribution to GDP in Pakistan. Pakistan Vis. (2019) 20:175.

[47] Daniels PW O'Connor KB Hutton TA. The planning response to urban service sector growth: an international comparison. Growth Change. (2010) 22:3–26. 10.1111/j.1468-2257.1991.tb00560.x

[48] Hanlon M Burstein R Masters SH Zhang R. Exploring the relationship between population density and maternal health coverage. BMC Health Services Res. (2012) 12:416. 10.1186/1472-6963-12-416 23170895 PMC3511226

[49] Kubota Y Heiss G MacLehose RF Roetker NS Folsom AR. Association of educational attainment with lifetime risk of cardiovascular disease: the atherosclerosis risk in communities study. JAMA Intern Med. (2017) 177:1165–72. 10.1001/jamainternmed.2017.1877 28604921 PMC5710437

[50] Levin A Lin CF Chu CJ. Unit root tests in panel data: asymptotic and finite-sample properties. J Econometr. (2002) 108:1–24. 10.1016/S0304-4076(01)00098-7

[51] Im KS Pesaran MH Shin Y. Testing for unit roots in heterogeneous panels. J Econometr. (2003) 115:53–74. 10.1016/S0304-4076(03)00092-7

[52] Swift R. The relationship between health and GDP in OECD countries in the very long run. Health Econ. (2011) 20:306–22. 10.1002/hec.1590 20217835

[53] Su CW Khan K Tao R Umar M. A review of resource curse burden on inflation in venezuela. Energy. (2020) 204:1–11. 10.1016/j.energy.2020.117925

